# Fertilizer ^15^N balance in a soybean–maize–maize rotation system based on a 41-year long-term experiment in Northeast China

**DOI:** 10.3389/fpls.2023.1105131

**Published:** 2023-01-30

**Authors:** Jian Dai, Hailong Gui, Feng Shen, Yuying Liu, Minsong Bai, Jinfeng Yang, Houjun Liu, Peiyu Luo, Xiaori Han, Kadambot H. M. Siddique

**Affiliations:** ^1^ Agricultural Resources and Environment Mobile Station, College of Land and Environment, Shenyang Agricultural University, Shenyang, Liaoning, China; ^2^ National Engineering Research Center for Efficient Utilization of Soil and Fertilizer Resources/Monitoring & Experimental Station of Corn Nutrition and Fertilization in Northeast Region, Ministry of Agriculture and Rural Affairs, Shenyang, Liaoning, China; ^3^ Food Science College, Shenyang Agricultural University, Shenyang, Liaoning, China; ^4^ The UWA Institute of Agriculture, The University of Western Australia, Perth, WA, Australia

**Keywords:** fertilizer N fate, long-term experiment, soybean-maize-maize rotation, N recovery, soil residual N, N losses

## Abstract

Global awareness of the need to enhance crop production and reduce environmental issues associated with nitrogen (N) fertilizer has increased. However, studies on how the N fate changed with manure addition are still limited. To explore efficient fertilization management for an improved grain yield, N recovery efficiency, and reduced N residual in the soil or that unaccounted for, a field 15N micro-plot trial in a soybean–maize–maize rotation was conducted to evaluate the effect of fertilization regimes on soybean and maize yields and the fertilizer N fate in the plant–soil system during 2017–2019 within a 41-year experiment in Northeast China. Treatments included chemical N alone (N), N and phosphorus (NP), N, P, and potassium (NPK), and those combined with manure (MN, MNP, and MNPK). Application of manure increased grain yield, on average, by 153% for soybean (2017) and 105% and 222% for maize (2018 and 2019) compared to no manure, with the highest at MNPK. Crop N uptake and that from labeled ^15^N-urea also benefited from manure addition, mainly partitioned to grain, and the average ^15^N-urea recovery was 28.8% in the soybean season with a reduction in the subsequent maize seasons (12.6%, and 4.1%). Across the three years, the fertilizer ^15^N recovery ranged from 31.2–63.1% (crop) and 21.9–40.5% (0–40 cm soil), with 14.6–29.9% unaccounted for, including N losses. In the two maize seasons, manure addition significantly increased the residual ^15^N recovery in crop attributed to the enhancing ^15^N remineralization, and reduced that in soil and unaccounted for compared to single chemical fertilizer, with MNPK performing the best. Therefore, applying N, P, and K fertilizers in the soybean season and NPK combined with manure (13.5 t ha^–1^) in the maize seasons is a promising fertilization management strategy in Northeast China and similar regions.

## Graphical Abstract

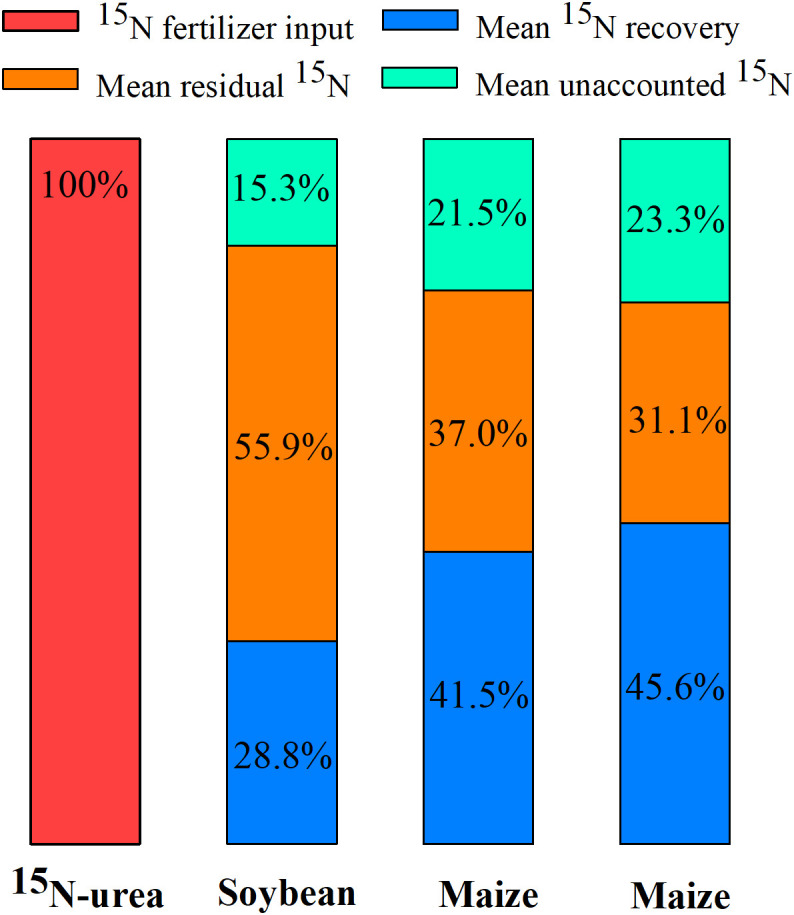

## Introduction

Increased fertilizer inputs, especially synthetic N fertilizer is a widely used method to increase crop production (Zhang et al., 2013; Ladha et al., 2016; ). However, less than 40% of the applied N was taken up by crops in China, resulting in severe consequences for food production and the environment (Lassaletta et al., 2014; Liu et al., 2021). The low N use efficiency resulted from the increased N fertilizer rate and even overuse (Ju et al., 2009; Hou et al., 2017; ). As the N rate increased, crop grain yields increased slightly or decreased, but inorganic N residual in the soil increased drastically, with elevated N losses (Dai et al., 2015). Eickhout et al. (2006) reported that total reactive N losses worldwide would increase from 1995–2030 with the growing intensive agriculture. Therefore, improving fertilization management is pivotal for enhancing crop production and N use efficiency, and reducing N losses.

Integrating manure into fertilizer management has a long history in China, and is an effective strategy for reducing chemical fertilizer use and sustaining agricultural production and soil fertility (Liang et al., 2013; Zhao et al., 2016; Cai et al., 2019; ). For example, an investigation of 153 field experiments revealed that adding manure enhanced crop yields by 8.5–14.2 Mg ha^–1^ (Chen et al., 2014a) and by 75% for maize on the North China Plain (Yang et al., 2015). In a similar region, replacing 30% of N fertilizer with manure (cow waste) significantly increased N uptake and N use efficiency compared to single chemical fertilizer (Zhang et al., 2016). The application of manure can not only improve crop production, but can also increase fertilizer N retention in the soil through immobilization and thus decreased the environmental risk associated with N losses, i.e., NH_3_ volatilization, N_2_O emissions, and nitrate leaching (Zhou et al., 2009; Li et al., 2020; Li et al., 2021a). However, as a result of increase in the chemical fertilizers, the using amount of manures has markedly decreased in recent years. Therefore, application of manure combined with inorganic fertilizers is currently recommended and still a challenge in the agricultural production in China.

Manure amendment influenced the fate of fertilizer N in the plant-soil system, either directly by enhancing fertilizer N uptake, or indirectly by changing soil properties such as pH, organic matter etc., which in turn favored N transformation and affected chemical fertilizer N residual in soil and loss (Gai et al., 2018; Wang et al., 2019). Residual N in the soil was mainly in the organic form, which could be re-mineralized to supply N for successive crops (Liang et al., 2013; Guo et al., 2021; ). In arid Saharan Morocco, a ^15^N-tracer field experiment reported a fertilizer ^15^N use efficiency of 33.1% in the first wheat season, with a soil residual ^15^N recovery of 2.4% in the succeeding season (Ichir et al., 2003). A meta-analysis determined that the average proportion of soil residual ^15^N recovered in the first subsequent crop was 5% (VSN International, 2015) compared with 6.2–7.0% in the following wheat season in Northwest China (Xia et al., 2018), 2.9% in the second grass season (Rocha et al., 2019), and 14.5% in the next legume season (Gathumbi et al., 2003). Variations in the residual N recovery are attributed to climatic conditions, cropping system, soil type, and fertilization management (Dourado-Neto et al., 2010; He et al., 2020).

In Northeast China, maize and soybean are the staple cereal cash crops, where maize–soybean is the main rotation system. However, the fate of fertilizer N in the plant–soil system and recovery of soil residual N in the subsequent crops have not been well quantified, especially in diverse crop rotations with combined chemical fertilizers and manure regimes. In this study, ^15^N-labeled micro-plots were established within a 41-year long-term field experiment to (1) quantify the fate of ^15^N-urea in the soybean season (^15^N allocation in each organ, residue in soil, and unaccounted ^15^N, including N losses); (2) identify the residual effect of fertilizer ^15^N on crop N uptake in the two subsequent maize seasons; (3) investigate the balance of fertilizer ^15^N in the soil–plant system under a soybean–maize–maize rotation (2017–2019), and thus explore the best fertilization management strategy to obtain high grain yields and N use efficiencies with low environmental risk.

## Materials and methods

### Study site

The 41-year experiment commenced in 1979 at the Long-Term Fertility Experimental Station of Shenyang Agricultural University (40°48′ N, 123°33′ E), Liaoning, China, with a subhumid climate. The average annual temperature from 1979 to 2019 was 8.5°C (21.2°C from May to September), and annual precipitation was 681 mm, with 538 mm during the crop growing season. The soil is a Haplic Luvisol (FAO, 1998), which contained 48% sand, 29% silt, and 23% clay in the 0–20 cm soil layer in 1979 with 1.18 g cm^–3^ bulk density, 15.9 g kg^–1^ organic matter, 0.8 g kg^–1^ total N, 105.5 mg kg^–1^ alkali-hydrolyzable N, 6.5 mg kg^–1^ Olsen-P, 97.9 mg kg^–1^ available K, and pH 6.5.

### Experimental design and field management

#### Field experiment

The field experiment was implemented with a soybean–maize–maize rotation, as described by Zhang et al. (2021). The six treatments included chemical fertilizer only (N, NP, and NPK) and manure combined with chemical fertilizer (MN, MNP, and MNPK). In the 41-year experiment, soybean received 30 kg N ha^–1^ as urea, 90 kg P_2_O_5_ ha^–1^ as triple superphosphate, 90 kg K_2_O ha^–1^ as potassium sulfate, and 13.5 t ha^–1^ organic fertilizer as pig manure in dry weight, while maize received 120 kg N ha^–1^, 60 kg P_2_O_5_ ha^–1^, 60 kg K_2_O ha^–1^, and 13.5 t ha^–1^ (dry weight) manure (**Table S1**). The average concentration of nutrients in pig manure is available in Li et al. (2021b). The application of pig manure to soybean crops ceased from 1992 onwards due to decreasing grain yields. The chemical fertilizer and manure were broadcast evenly over the soil before sowing and plowed into the top 20 cm of soil using a rotavator.

Soybean (Liaodou15) and maize (Dongdan6531) were sown at 15 × 10^4^ and 6 × 10^4^ plants ha^–1^ in early May and harvested in late September or early October in 10 m × 16 m plots with rows spaced 60 cm apart. No supplemental irrigation was provided. Pest and weed control was consistent with local practices.

#### ^15^N-labeled micro-plots

To assess the fate of fertilizer N in the soybean–maize–maize rotation, a ^15^N-labeled plot study was undertaken over three growing seasons (2017–2019). Before soybean sowing in 2017, micro-plots (0.8 m × 0.6 m) were established in triplicate in each plot. The micro-plots comprised a 50 cm high polyvinyl-chloride frame, of which 10 cm was above the soil surface. Each micro-plot contained eight soybean plants in 2017 and three maize plants in 2018 and 2019. ^15^N-labeled urea with 20.2 atom% (Shanghai Chem-Industry Institute) was mixed with P and K into the 15 cm of topsoil from each micro-plot and then returned to the corresponding micro-plot. In the two subsequent maize seasons in 2018 and 2019, urea (unlabeled) mixed with P and K and pig manure were applied using the abovementioned procedure. All management practices matched those in the macro-plots. **Table S2** lists the chemical properties of the top 40 cm soil before soybean seeding in 2017.

#### Sampling and analysis

Plant samples were collected from each micro-plot at harvest in each crop growing season. Soybean plants were separated into aboveground parts and roots, which were cut with a sickle at the stem and root joint. After air-drying, the aboveground parts were threshed, separated into straw (including stems and leaves), pods, and grain, and weighed. Soybean roots were dug out of each micro-plot using a spade and the roots left in the soil were picked out. The root samples were firstly washed with tap water to remove the attached soil and then with distilled water. Subsamples of each organ (roots, straw, pods, and grain) were oven-dried at 90°C for 30 min and then at 65°C for 48 h to calculate water content and dry weight. The oven-dried samples (roots, straw, pods, and grain) were ground and passed through a 0.5 mm sieve for total N and its ^15^N enrichment determination. Similarly, maize was cut at the stem base and partitioned into straw, bracts, cobs, grain, and roots, and weighed after being air-dried. Subsamples were dried, crushed, and sieved for further measurement.

Soil samples were randomly collected in each micro-plot using an auger at 0–20 and 20–40 cm depths before sowing and between two plants after harvest of soybean and maize (2017–2019). Soil samples from the two duplicate soil cores in each micro-plot were mixed and sealed in separate marked plastic bags and stored at 4°C for later determination of soil water, mineral N, and ^15^N contents. Subsoil samples from the same depth and micro-plot were air-dried, ground, and sieved (equivalent to 0.15 mm) for total N and ^15^N abundance determination. Soil mineral N ( ${\text{NO}}_{3}^{-}-\text{N}$ and ${\text{NH}}_{4}^{+}-\text{N}$ ) was extracted using 50 mL of 2 mol L^–1^ KCl (1:5 soil:solution) and measured with a high-resolution digital colorimeter AutoAnalyzer3 (AA3, SEAL Company, Germany); their ^15^N abundance was determined using the diffusion method (Keeney and Nelson, 1983; Sebilo et al., 2004). Plant and soil total N concentrations and their ^15^N enrichment were analyzed using an automated C/N analyzer isotope ratio mass spectrometer (Elementar III-IRMS). Plant and soil samples from a control macro-plot were used to assess natural ^15^N abundance.

### Calculations

#### Crop N Uptake and N Uptake Derived from ^15^N-urea

Crop N uptake was calculated as follows:


(1) $$\begin{array}{c} \text{Crop N uptake }\left({\text{kg N ha}}^{–1}\right)\\ \;=\sum (\text{N concentration}\times \text{dry weight})\;/\;1000 \end{array}$$

where N concentration (g N kg^–1^) refers to that of soybean roots, straw, pods, and grain or maize roots, straw, bracts, cobs, and grain; dry weight is the biomass (kg ha^–1^) of each organ, and 1000 is the conversion coefficient.

The percentage of ^15^N-urea taken up by soybean or maize (Ndff) was calculated as (Hu et al., 2019):


(2) Ndff (%) = (a–b)/(c–b)×100

where a and b are the atom% of ^15^N in the plant sample in the ^15^N-labeled treatment and control treatment, respectively, and c is the atom% of ^15^N in urea (20.2%).

Crop N uptake derived from ^15^N-urea during the soybean season and soil residual ^15^N in the two subsequent maize seasons (Ndff_plant_) and the fertilizer ^15^N recovery rate in plants were calculated according to the following equations (de Oliveira Silva et al., 2017):


(3) $${\text{Ndff}}_{\text{plant}}\;({\text{kg N ha}}^{–1})\;=\sum \left(\text{Nuptake}\times \text{plant Ndff}\right)$$

where N uptake (kg N ha^–1^) is the total N uptake in soybean roots, straw, pods, and grain or maize roots, straw, bracts, cobs, and grain, and plant Ndff (%) is the percentage of ^15^N-urea taken up in each organ.


(4) $$\text{Recovery rate }\left(\%\right)=\left(3\right)/\text{N fertilizer rate }\left({\text{kg N ha}}^{–1}\right)\;\times \;100$$

The ^15^N harvest index (^15^NHI, %) in the three growing seasons was calculated as the ratio of grain ^15^N uptake to crop ^15^N uptake:


(5) $${\;}^{15}\text{NHI}\;(\%)={\text{Grain}}^{15}\text{N uptake }\left({\text{kg N ha}}^{–1}\right)/(3)\times 100$$

#### Residual ^15^N-urea in soil

The percentage of ^15^N-urea remaining in soil (Ndff) was calculated as per Eq. (2). The amount of soil total residual N derived from ^15^N-urea (Ndff_soil_) and its residual rate in 0-20 and 20-40 cm soil layers were calculated as


(6) $$\begin{array}{c} {\text{Ndff}}_{\text{soil }}\left({\text{kg N ha}}^{–1}\right)\\ =\text{soil total N concentration }\left({\text{mg N kg}}^{–1}\right)\times \text{soil depth increment }\left(\text{cm}\right)\\ \times \text{soil bulk density }\left({\text{g cm}}^{–3}\right)/10\times \text{soil Ndff }\left(\%\right) \end{array}$$

(7) $${\;}^{15}\text{N residual rate }\left(\%\right)=\left(6\right)/\text{N fertilizer rate }\left({\text{kg N ha}}^{–1}\right)\times 100$$

The amount of soil mineral N (Ndff_min_) and organic N (Ndff_org_) derived from ^15^N-urea in each soil layer were calculated as:


(8) $$\begin{array}{c} {\text{Ndff}}_{\text{min}}\;({\text{kg N ha}}^{–1})\\ =\text{soil mineral N concentration}\;({\text{mg N kg}}^{–1})\times \text{soil depth increment }\left(\text{cm}\right)\\ \times \text{soil bulk density}\;({\text{g cm}}^{–3})/10\times {\text{soil Ndff}}_{\text{min}}\;(\%) \end{array}$$

(9) $${\text{Ndff}}_{\text{org }}\left({\text{kg N ha}}^{–1}\right)=\left(6\right)\;–\;\left(8\right)$$

#### Unaccounted ^15^N

The unaccounted N derived from ^15^N-urea (including fertilizer N losses, i.e., gas emissions, leaching, and to deeper layers) and the corresponding rate were calculated as:


(10) $${\text{Unaccounted}}^{15}\text{N }\left({\text{kg N ha}}^{–1}\right)=\text{N fertilizer rate}\;({\text{kg N ha}}^{–1})–\left(3\right)–\left(6\right)$$

(11) $${\text{Unaccounted}}^{15}\text{N rate }\left(\%\right)=\left(10\right)/\text{N fertilizer rate }\left({\text{kg N ha}}^{–1}\right)\;\times 100$$

### Statistical analysis

Statistical analysis was performed using the SAS software package (Version 9.2). A one-way ANOVA was used to test the effect of different fertilization treatments on total N uptake and that derived from ^15^N-urea by soybean and maize, ^15^N residual in soil, and unaccounted ^15^N losses across the three rotation years. Significant differences between treatments were compared using the least significant difference method at the 0.05 probability level.

## Results

### Growing conditions

**Figure 1:**
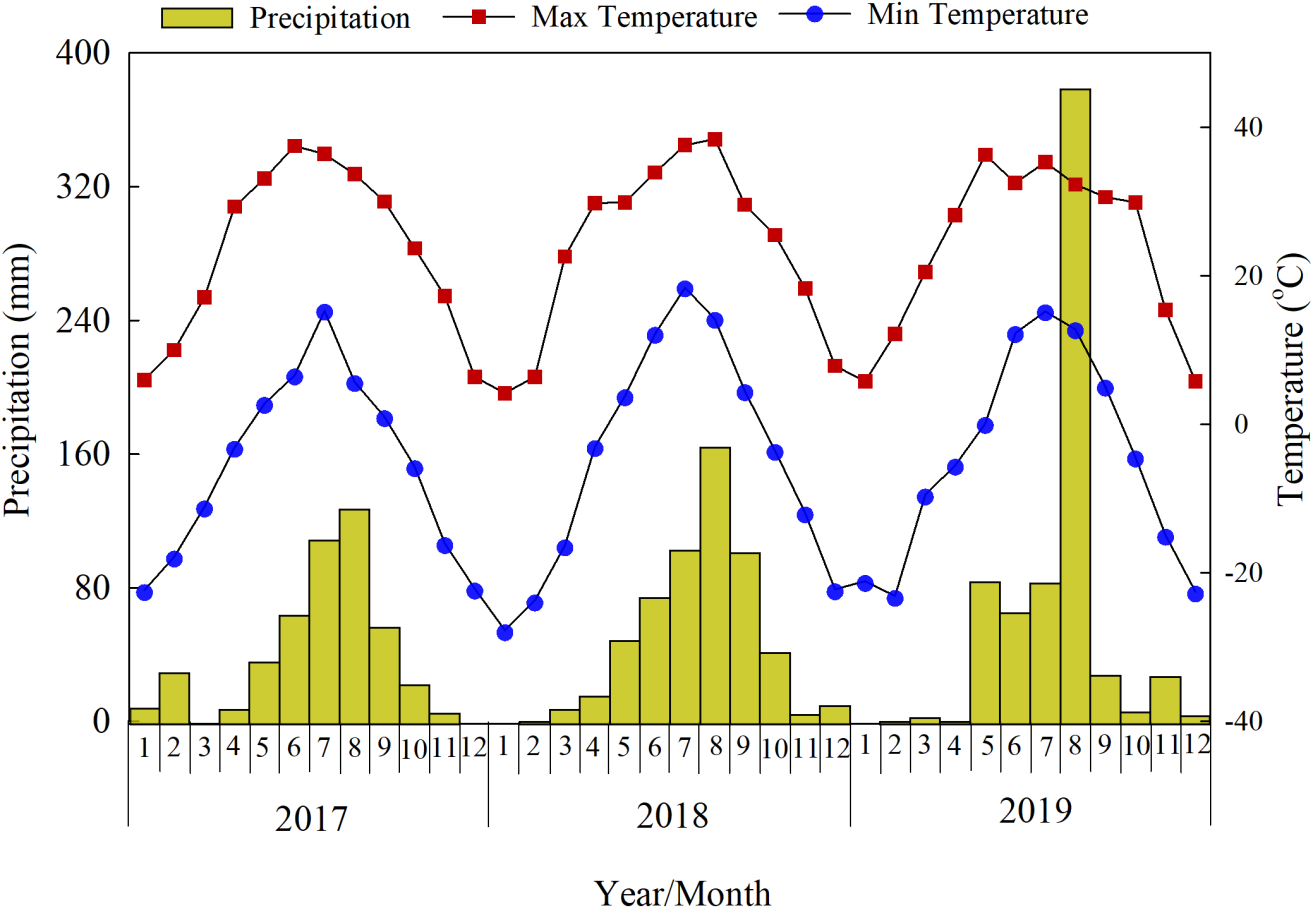
Monthly precipitation, maximum and minimum temperatures across the soybean–maize–maize growing seasons (2017–2019). Precipitation was measured in the experimental field with a Rain Gauge (adcon). Maximum and minimum temperatures come from the China National Meteorological Administration.

At the experimental site, the annual precipitation was 477, 583, and 691 mm in 2017, 2018, and 2019, with 83%, 85%, and 93% (averaged 87%) occurring during the crop growing seasons (**Figure 1**). The trends in monthly maximum and minimum temperatures were similar across years, with maximum temperatures of 37.5, 38.4, and 36.3°C and minimum temperatures of –22.3, –27.7, and –23.1°C in 2017, 2018, and 2019, respectively.

### Soybean and maize grain yield

**Figure 2:**
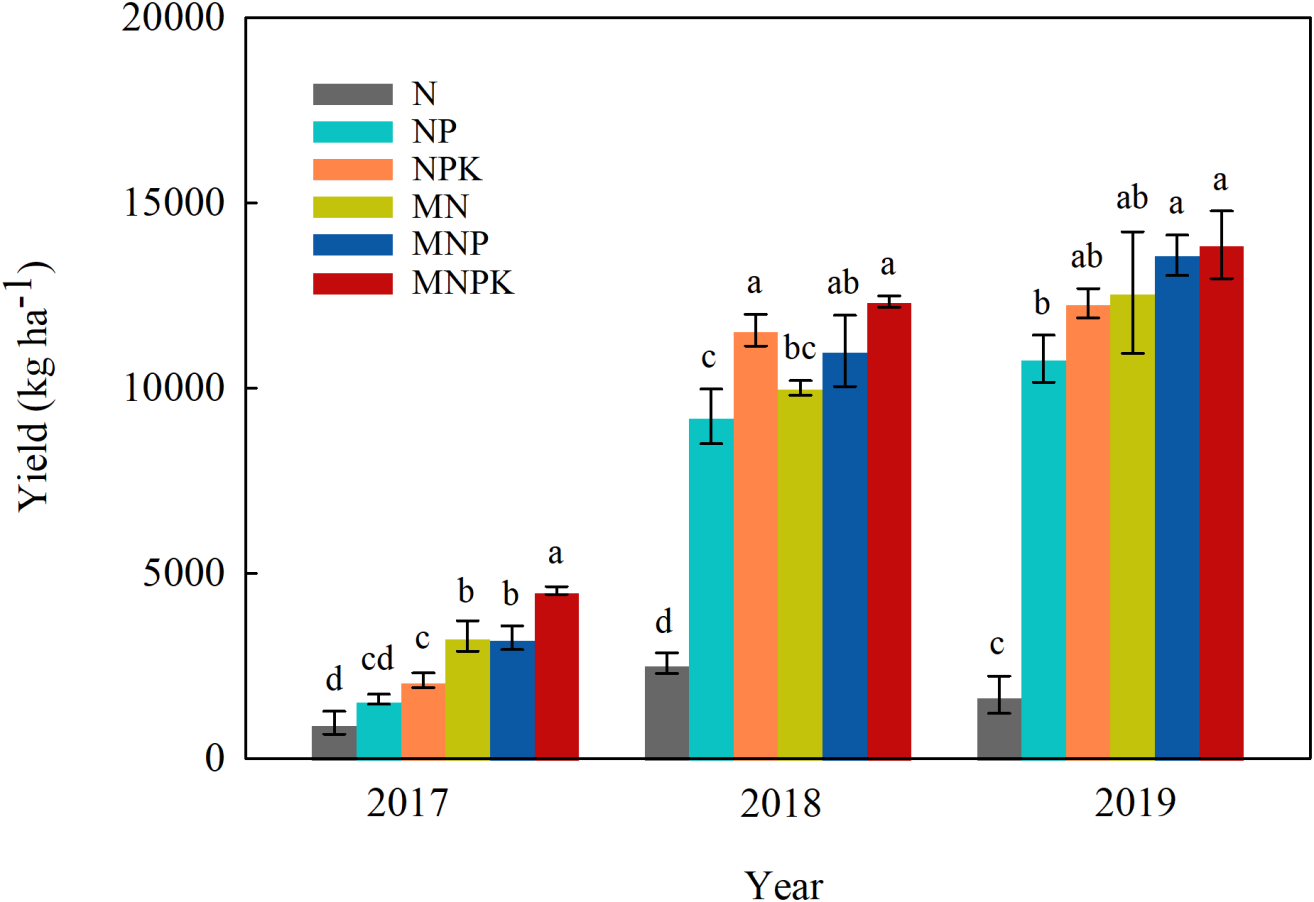
Grain yields in different fertilization treatments in the 2017–2019 soybean–maize–maize growing seasons. Bars represent standard errors (n = 3 replicates). Different lowercase letters denote significant differences (*p*< 0.05) among treatments each year according to the LSD test.

Soybean and maize grain yields varied between treatments (**Figure 2**). In the soybean season (2017), the combined chemical fertilizer and manure treatments significantly increased grain yield by 153% on average, relative to chemical fertilizer alone (241% at MN vs N, 103% at MNP vs NP, and 114% at MNPK vs NPK), more so at MNPK (yield being 4544 kg ha^–1^). The yield trends in the two succeeding maize seasons were similar to soybean, with the highest yields at MNPK (12336 and 13868 kg ha^–1^ in 2018 and 2019, respectively). The MN, MNP, and MNPK treatments had 288%, 19%, and 7% higher maize grain yields than the N, NP, and NPK in 2018, respectively, with corresponding increases of 628%, 26%, and 13% in 2019.

### Soybean and maize N uptake and N uptake derived from fertilizer ^15^N

**Figure 3:**
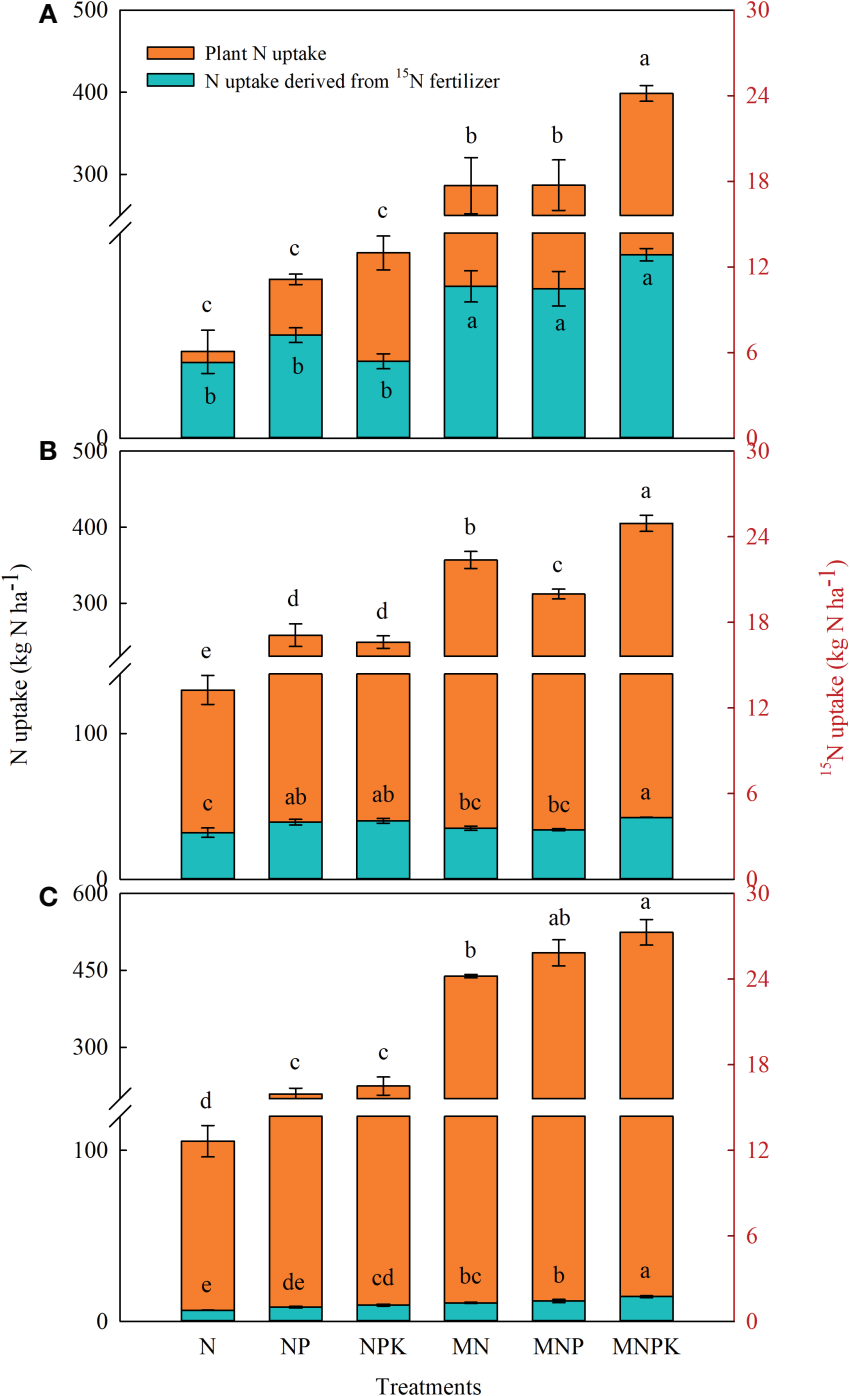
Plant N uptake and N uptake derived from ^15^N fertilizer across the soybean–maize–maize rotation in 2017 **(A)**, 2018 **(B)**, and 2019 **(C)**. Bars represent standard errors (n = 3 replicates). Different lowercase letters indicate significant differences (*p*< 0.05) among treatments in each year according to the LSD test.

The combined chemical fertilizer and manure treatments significantly enhanced soybean and maize N uptake relative to the sole chemical fertilizer treatments (**Figure 3**). In 2017, soybean total N uptake ranged from 44.4–398.5 kg N ha^–1^, of which 5.3–12.9 kg N ha^–1^ came from ^15^N-urea, with the maximum amounts at MNPK. The ratio of plant N derived from ^15^N-urea to total N uptake decreased from 12.0% at N to 3.2% at MNPK. In the two subsequent maize seasons, plant N uptake ranged from 130.1–405.1 kg N ha^–1^ in 2018 and 105.4–524.2 kg N ha^–1^ in 2019, of which 3.3–4.3 and 0.8–1.7 kg N ha^–1^ came from soil residual ^15^N from the soybean season. Maize N uptake from the soil residual ^15^N contributed 1.0–2.5% and 0.3–0.8% to total N uptake in 2018 and 2019, respectively. As with the soybean season, the MNPK treatment had the highest values, which significantly differed from the other treatments.

**Figure 4:**
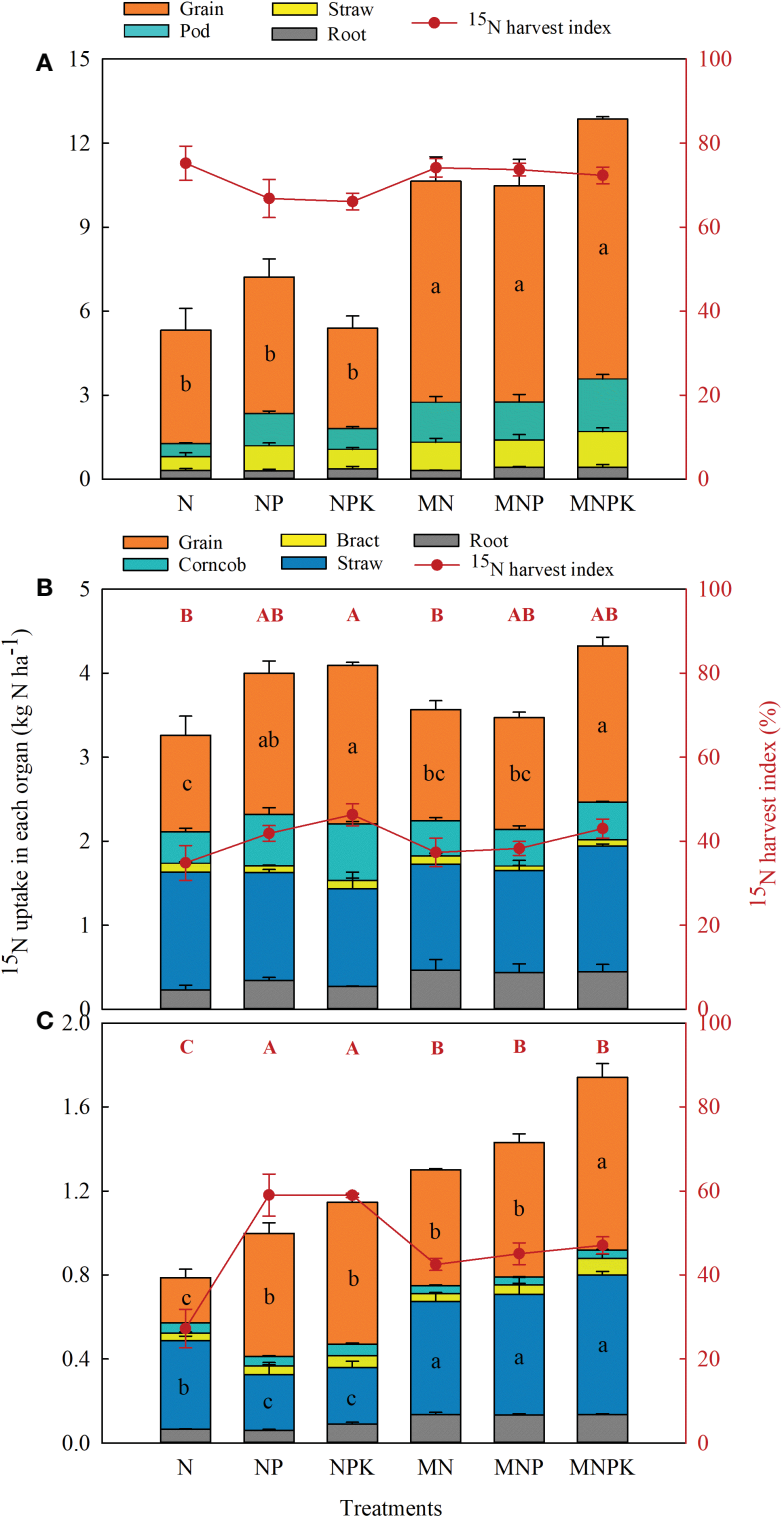
Allocation of fertilizer ^15^N in plant organs of soybean and maize and ^15^N harvest index (^15^NHI) in 2017 **(A)**, 2018 **(B)**, and 2019 **(C)**. Different lowercase and uppercase letters indicate significant differences (*p*< 0.05) in ^15^N uptake and ^15^NHI, respectively, among treatments each year according to the LSD test.

The distribution of ^15^N uptake in plant organs among treatments followed similar trends each season (**Figure 4**). In the soybean season, ^15^N uptake was partitioned mainly to grain, with the highest at MNPK (9.3 kg N ha^–1^), and ^15^NHI ranged from 66.1–75.2% across all treatments (**Figure 4A**). The combined chemical fertilizer and manure treatments significantly increased ^15^N uptake in grain by 104.2% on average, with no significant effect on ^15^NHI, relative to the chemical fertilizer only treatments. The distribution of ^15^N-urea in pods, straw, and roots accounted for 13.2%, 10.5%, and 4.6% of the total ^15^N uptake on average, with no significant differences between treatments. In the first subsequent maize season (2018), the NPK and MNPK recovered 1.88 and 1.86 kg ha^–1^ soil residual ^15^N from the soybean season for distribution to grain, with ^15^NHI of 46.3% and 43.0%, respectively. The ratios of ^15^N uptake in cobs, bracts, straw, and roots to total uptake were 13.0%, 2.3%, 34.8%, and 9.7%, respectively. In the second subsequent maize season (2019), the recovery of soil residual ^15^N declined but followed a similar trend to 2018. The average ^15^NHI was 46.6%, with ratios ^15^N uptake in cobs, bracts, straw, and roots to total uptake of 3.9%, 4.1%, 37.2%, and 8.3%, respectively.

### Fertilizer ^15^N residual in soil

**Figure 5:**
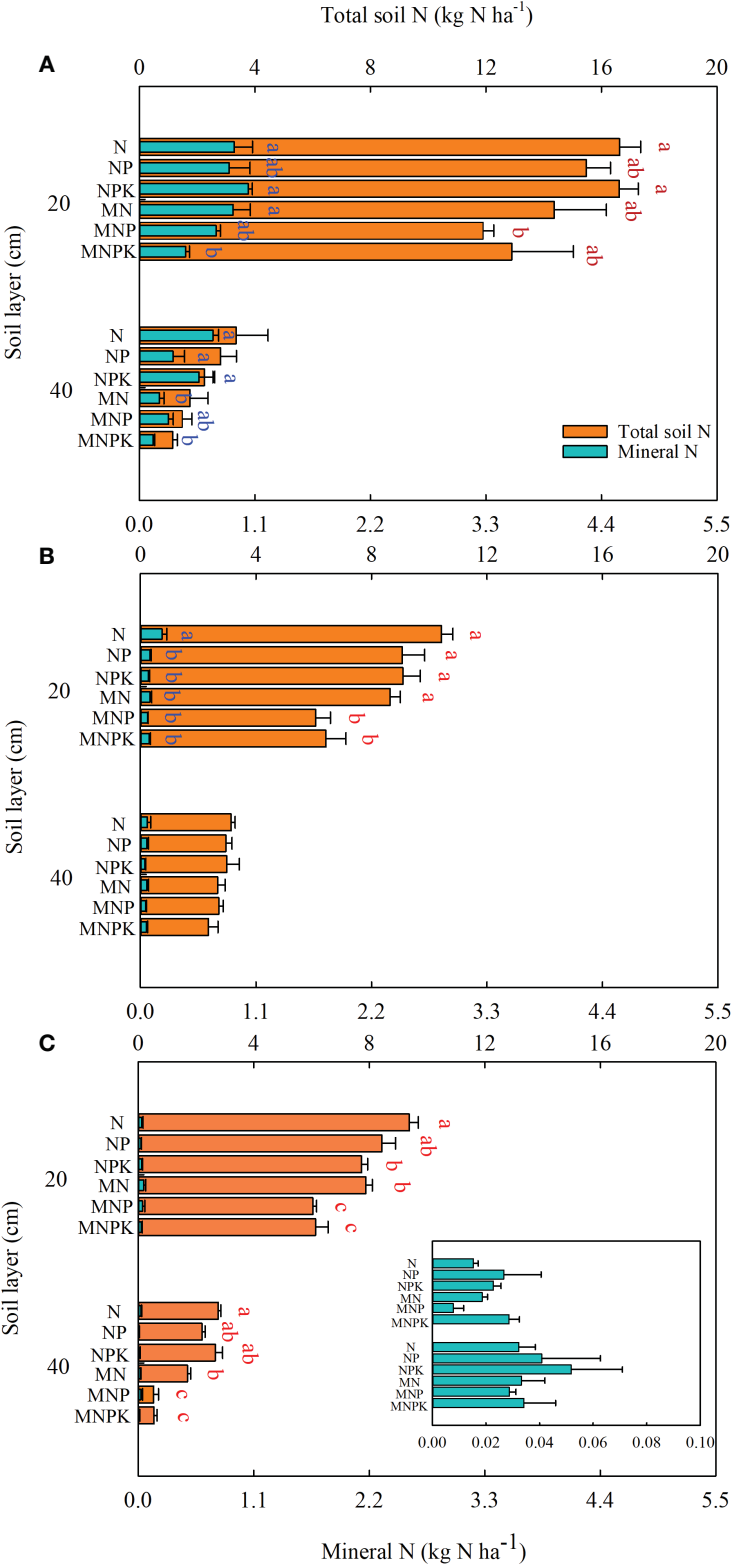
Residual fertilizer ^15^N as total soil N and mineral N in the 0–40 cm soil layer at soybean and maize harvest in 2017 **(A)**, 2018 **(B)**, and 2019 **(C)**. Different lowercase letters indicate significant differences (*p*< 0.05) in total soil N (red letters) and mineral N (blue letters) among treatments each year in the 0–20 cm and 20–40 cm soil layers according to the LSD test.

Of the 30 kg N ha^–1 15^N-urea applied in the soybean season, 13.4–20.0 kg N ha^–1^ remained in the 0–40 cm soil layer at harvest, with 83.2–91.8% in the top 20 cm (**Figure 5A**). Chemical fertilizer alone resulted in more ^15^N residual in the top 40 cm soil with no significant difference compared to treatments with manure. The residual ^15^N-urea was present mainly as organic N, accounting for 58.6% of the added ^15^N-urea in the chemical fertilizer only treatments and 45.4% in the chemical fertilizer combined with manure treatments, compared with 4.9% and 3.0% for mineral N, respectively. At maize harvest in the first subsequent year, the top 40 cm soil contained 8.8–13.6 kg N ha^–1^ derived from ^15^N-urea, with 69.1–76.9% in the 0–20 cm soil (**Figure 5B**). Similarly, 31.6% and 41.2% of the preceding applied ^15^N fertilizer remained in organic form in the 0–40 cm soil profile with and without manure, respectively, compared with 0.5% and 0.6% for mineral N. In the second succeeding maize year, the top 40 cm soil contained 6.7–12.2 kg N ha^–1^ derived from ^15^N-urea, with a significant decrease with manure application (**Figure 5C**), with 74.3–91.9% distributed in the top 20 cm layer. The ratios of organic N to applied ^15^N-urea were 25.2% and 36.7% with and without manure, compared with 0.2% and 0.2% for mineral N.

### Total recovery of fertilizer ^15^N and balance in soybean–maize–maize system

The chemical fertilizer combined with manure treatments significantly increased soybean N uptake from applied ^15^N-urea by 94.6% compared with the chemical fertilizer only treatments (**Table 1**). In the soybean season, the fertilizer ^15^N recovery was 28.8% on average. In the two succeeding maize seasons, 3.3–4.3 kg N ha^–1^ and 0.8–1.7 kg N ha^–1^ of the soil residual ^15^N from the soybean season was recovered, accounting for 10.9–14.4% (12.6% average) and 2.6–5.8% (4.1% average) of theapplied ^15^N-urea. Across the soybean–maize–maize rotation system, 31.2–63.1% of the initial applied fertilizer ^15^N was recovered, with 21.9–40.5% remaining in the 0–40 cm soil and 20.2–31.3% in the top 20 cm soil. During the 3-year rotation, 14.6–29.9% of the applied ^15^N-urea was unaccounted for (including ^15^N losses), which decreased by 26.9% with manure addition. The highest recovery rate (63.1%) and lowest residual (22.3%) and unaccounted (14.6%) rates were found at MNPK treatment.

**Table 1:** Uptake of applied ^15^N fertilizer, residual ^15^N in soil, and unaccounted ^15^N in the 2017 soybean, 2018 maize, and 2019 maize seasons in a 3-year rotation in Northeast China.

Treatment	Crop ^15^N uptake (kg N ha^–1^)				Soil residual ^15^N after 3 years (kg N ha^–1^)			Unaccounted ^15^N in the system (kg N ha^–1^)
	Soybean 2017	Maize 2018	Maize 2019	Total 2017-2019	0–20 cm	20–40 cm	0–40 cm	
N	5.3 ± 0.7b	3.3 ± 0.3c	0.8 ± 0.01e	9.4 ± 0.7d	9.4 ± 0.3a	2.8 ± 0.09a	12.2 ± 0.2a	8.5 ± 0.9a
NP	7.2 ± 0.5b	4.0 ± 0.1ab	1.0 ± 0.06de	12.2 ± 0.2c	8.4 ± 0.4ab	2.2 ± 0.1b	10.6 ± 0.5b	7.1 ± 0.5ab
NPK	5.4 ± 0.5b	4.1 ± 0.1ab	1.1 ± 0.07cd	10.6 ± 0.4cd	7.7 ± 0.2b	2.7 ± 0.2ab	10.4 ± 0.3bc	9.0 ± 0.6a
MN	10.6 ± 1.0a	3.6 ± 0.1bc	1.3 ± 0.04bc	15.5 ± 1.0b	7.9 ± 0.2b	1.7 ± 0.1c	9.6 ± 0.2c	4.9 ± 0.9bc
MNP	10.5 ± 1.2a	3.5 ± 0.07bc	1.4 ± 0.11b	15.4 ± 1.1b	6.1 ± 0.1c	0.5 ± 0.1d	6.6 ± 0.1d	8.0 ± 1.2a
MNPK	12.9 ± 0.4a	4.3 ± 0.01a	1.7 ± 0.06a	18.9 ± 0.5a	6.1 ± 0.4c	0.5 ± 0.1d	6.7 ± 0.3d	4.4 ± 0.3c
Ratio to fertilizer N application rate (%)								
N	17.7 ± 2.5b	10.9 ± 1.0c	2.6 ± 0.04e	31.2 ± 2.5d	31.3 ± 1.0a	9.2 ± 0.3a	40.5 ± 0.9a	28.3 ± 3.1a
NP	24.1 ± 1.7b	13.3 ± 0.6ab	3.3 ± 0.2de	40.7 ± 0.9c	28.1 ± 1.5ab	7.4 ± 0.3a	35.5 ± 1.7b	23.8 ± 1.9ab
NPK	18.0 ± 1.7b	13.6 ± 0.5ab	3.8 ± 0.2cd	35.4 ± 1.3cd	25.8 ± 0.6b	8.9 ± 0.8a	34.7 ± 1.1bc	29.9 ± 2.2a
MN	35.5 ± 3.6a	11.9 ± 0.5bc	4.3 ± 0.1bc	51.7 ± 3.4b	26.3 ± 0.7b	5.7 ± 0.3b	31.9 ± 0.6c	16.4 ± 3.2bc
MNP	34.9 ± 4.0a	11.6 ± 0.2bc	4.8 ± 0.3b	51.3 ± 3.7b	20.2 ± 0.4c	1.8 ± 0.5c	21.9 ± 0.4d	26.8 ± 4.0a
MNPK	42.9 ± 1.4a	14.4 ± 0.05a	5.8 ± 0.2a	63.1 ± 1.6a	20.5 ± 1.4c	1.8 ± 0.3c	22.3 ± 1.1d	14.6 ± 1.0c

N: chemical N fertilizer; NP: chemical N and P fertilizer; NPK: chemical N, P, and K fertilizer; MN: chemical N fertilizer combined with manure; MNP: chemical N and P fertilizer combined with manure; MNPK: chemical N, P, and K fertilizer combined with manure. Values are means ± standard error (n = 3 replicates). Different lowercase letters in each column indicate significant differences (p< 0.05) in fertilizer ^15^N uptake, residual ^15^N in soil, and unaccounted ^15^N among treatments in the soybean–maize–maize rotation according to the LSD test.

## Discussion

### Grain yield and crop N uptake

The combined application of chemical and organic fertilizers increased crop yield compared to chemical fertilizer alone (**Figure 2**), as reported elsewhere (Chivenge et al., 2011; Doan et al., 2015; Zhang et al., 2018). In our study, grain yields significantly increased at treatments with manure in the soybean season, despite not being applied since 1992, which could be due to the increased soil organic matter and nutrient availability (**Table S2**). Similarly, a ^15^N study in Northwest China reported that soils supplied with MNPK had higher wheat grain yields than those supplied with NPK for the same amount of N applied (Liang et al., 2013), as did another study in the Tai Lake region (Pan et al., 2009). In maize seasons, grain yields were more responsive to chemical fertilizer plus manure than chemical fertilizer alone with reduced difference between manure treatments due to the extra nutrient addition. Besides, after long-term fertilization with chemical fertilizer alone, the soil had lower buffering capacity of nutrients and water conservation and supply than that with manure application (**Table S2**) (Diacono and Montemurro, 2010; Oldfield et al., 2019). However, another study reported no significant effect on maize yield for the same N rate, replacing 50% synthetic N fertilizer with manure in Northeast China (Chen et al., 2014b), and wheat yields even decreased by 14–20% in North China Plain (Sun et al., 2013).

Long-term addition with chemical fertilizer and manure benefited soybean and maize total N uptake and that derived from ^15^N-urea, particularly at MNPK (**Figure 3**), but did not alter its proportional distribution in plant organs. Plant N uptake from ^15^N fertilizer decreased in the two successive maize seasons, and totally accounted for 31.2–63.1% of the applied N in 2017, indicating that most of the ^15^N-urea remained in the soil or was lost. Previous studies reported comparable results (Wang et al., 2016a; Li et al., 2018; ). In contrast, the MNPK treatment had lower ^15^NHI than the NPK treatment due to the dilution effect, as the significant increase in total ^15^N uptake with manure decreased the ratio of N distributed in grain.

### ^15^N recovery efficiency in the soybean season

Co-application with manure significantly increased fertilizer ^15^N recovery by 94.6% compared with chemical fertilizer alone in the first soybean season, attributed to the enhancement in soil organic matter (22.4 g kg^–1^ with manure and 17.2 g kg^–1^ with chemical fertilizer) (**Table S2**), similar to previous studies (Liang et al., 2019; Quan et al., 2021). However, less than 50% of the ^15^N-urea was recovered in plant, with the highest at MNPK (42.9%). Similarly, another study searching numerous papers with ^15^N labeled fertilizer reported ^15^N recovery rates of 41% for maize, 32% for rice, and 37% for crops with small grains (Yan et al., 2019). Rocha et al. (2019) also reported that the seasonal ^15^N recovery for maize was 34%, with 46% residual in soil in the maize–pasture rotation system. It indicated that large amount of fertilizer N was residual in soil, a crucial N source for subsequent crops (Yang et al., 2011; Guo et al., 2021; ). Therefore, it is important to not only consider seasonal N fertilizer efficiency but also the recovery of soil residual N derived from fertilizer in succeeding seasons.

### Fertilizer ^15^N residual in the soil

For crop N uptake, 56–64% was derived from soil, with some converted from residual fertilizer N, biotic fixation, and N deposition (Gardner and Drinkwater, 2009; Dourado-Neto et al., 2010; ). Due to the overuse of N fertilizer in China, residual fertilizer N could be the largest contributor to the soil N pool (Zhang et al., 2015). At soybean harvest in 2017, 44.7% (MNP) to 66.7% (N) of the applied ^15^N-urea remained in the 0–40 cm soil layer (**Figure 5A**), comparable to irrigated (45–60%) and rainfed (49.8%) maize studies in China (Yang et al., 2011; Wang et al., 2016b; ). While, Guo et al. (2021) reported a lower fertilizer N residual rate (26.1–32.1%) due to the higher N use efficiency of maize (40–50%), and 30% in Shi et al. (2012) at an N rate of 210 kg N ha^–1^ with large N losses. In our study, the residual fertilizer ^15^N remained mainly in the top 20 cm soil though frequent rainfall occurred during the soybean and maize growing seasons. It might be attributed to the microbial immobilization and some adsorption of mineral N forms (presumably ${\text{NH}}_{4}^{+}$ ) in exchangeable sites of the topsoil, in line with other studies (Liu et al., 2015; Karwat et al., 2017; Hu et al., 2019; ). Inversely, higher fertilizer ^15^N accumulated in the 20–40 cm soil layer in North Central China, indicating that more N leaching occurred than in our study (Quan et al., 2020). In the second maize season in 2019, the 20–40 cm soil had more mineral N than the top 20 cm, attributed to the high precipitation in August (378.6 mm), which might have increased mineral N leaching. Like Li et al. (2021a), the combined use of manure and chemical fertilizer reduced the soil mineral N amount, potentially decreasing N leaching. Thus, fertilizer type, application method, soil properties, and climate influenced fertilizer N residual in soil. After three years, 21.9–40.5% of the labeled ^15^N fertilizer remained in the 0–40 cm soil depth, with the lower proportions at manure treatments, and was primarily in organic form, consistent with earlier studies (Stevens et al., 2005; Li et al., 2015; Van Meter et al., 2016). Subsequent crops can use the organic residual N through remineralization.

### Residual ^15^N recovery by the succeeding maize crops

Similar to other studies (Smith and Chalk, 2018; Yan et al., 2019), there was an incremental reduction in the recovery of soil residual ^15^N by successive crops. In the two maize seasons (2018 and 2019), 10.9–14.4% and 2.6–5.8% of the total applied ^15^N-urea was recovered, with the highest at MNPK. The reason is that the high soil fertility and improved physical and biological properties due to the long-term fertilization with chemical and organic fertilizers enhanced the release of soil residual organic ^15^N. A 60-year study of ^15^N tracer reported a soil residual ^15^N recovery of 5.4 ± 4.5% in the first subsequent season and<3% with impairing rate in the second or more successive seasons (Smith and Chalk, 2018). It indicated that the residual fertilizer N in organic form could remain in soil for a long time, which was gradually released and used by subsequent crops, with only 13 kg N ha^–1^ mineralized over 28 years (Sebilo et al., 2013). Exceptions to the above results were attributed to high N fertilizer rates and soil N residues, resulting in high N recoveries in the following cropping seasons (Ditsch et al., 1993; Ju et al., 2007; Guo et al., 2021; ). Across the three-year rotation, the MNPK treatment had the highest (63.1%) fertilizer N recovery, which could be because it had the highest ^15^N recovery in the first soybean season, and the manure stimulated microbial activity, enhancing ^15^N remineralization from the residual organic N (Edmeades, 2003; Ding et al., 2017; ) and thus increasing crop ^15^N uptake in the two maize seasons. Therefore, fertilization with manure plays a vital role in promoting fertilizer N recovery efficiency, most likely by improving soil fertility status.

### Unaccounted ^15^N during the three-year rotation

During the three-year rotation, 14.6–29.9% of the applied ^15^N-urea was unaccounted for, which might be related to processes of N removal to the deep soil layer and N losses, including gaseous emissions and leaching. Guo et al. (2021) reported that the proportion of unaccounted ^15^N fertilizer was 21.8–27.4%, mainly associated with N losses by volatilization, denitrification, and leaching as the soil residual N was monitored to 160 cm depth. Another study in dryland Northern China showed that 23% of total N losses came from ammonia volatilization (Ju, 2014) due to the high pH of the calcareous soil. In our study, ammonium ions were the main form in the soil, not free ammonia, with a pH below 7 (**Table S2**) (Overrein and Moe, 1967; Hayashi et al., 2011; ), thus ammonia volatilization could not be the dominant unaccounted ^15^N loss. Additionally, over 80% of the annual precipitation occurred during crop growing seasons in our study (**Figure 1**), which would result in more N losses by leaching. A global study on maize cropping systems consistenly reported 28% of unaccounted ^15^N under conventional management, with N rates ranging from 100–300 kg N ha^–1^ (Quan et al., 2021), whilea higher proportion of 37% was revealed in a meta-analysis with a larger range of N rates (average 114 kg N ha^–1^) (Gardner and Drinkwater, 2009). Application of manuredecreased the unaccounted ^15^N by 26.9% compared to the chemical fertilizer only treatments, mainly attributed to the long-term fertilization with manure plus inorganic fertilizer increasing soil C content, enhancing N immobilization and retaining organic N (Wang et al., 2019). In addition, the lower unaccounted ^15^N with manure was consistent with the higher total N recovery observed across the three years. Likewise, compost and straw application decreased soil mineral N contents, even at 80–100 cm depth, diminishing the risk of N leaching (Zhang et al., 2016). Therefore, raising the awareness of the benefits of manure application is crucial for increasing production and decreasing fertilizer N losses and thus environmental risks.

## Conclusion

Our results indicated that the addition of manure increased soybean and maize grain yield and benefited crop N uptake and that from labeled ^15^N-urea, which partitioned mainly to grain. On average, ^15^N-urea recovery was 28.8% in the soybean season and 12.6% and 4.1% in the two succeeding maize seasons. Across the three years, total fertilizer ^15^N recovery in the crop and 0–40 cm soil ranged from 31.2–63.1% and 21.9–40.5%, with 14.6–29.9% unaccounted for, including N losses. In the two subsequent maize seasons, manure addition significantly increased residual ^15^N recovery in the crop and reduced residual ^15^N in the soil and unaccounted for, with MNPK performing the best. Therefore, it showed that long-term NPK application in the soybean season, plus manure (i.e., 13.5 t ha^–1^) in the following maize seasons, can increase crop yield and fertilizer ^15^N recovery while reducing ^15^N residual in soil or losses and thus environmental risk; this strategy could be adopted widely in Northeast China and similar regions.

## Data availability statement

The original contributions presented in the study are included in the article/**Supplementary Material**. Further inquiries can be directed to the corresponding authors.

## Author contributions

PL supervised and edited the manuscript. XH designed the experiment and helped in writing and editing the manuscript. JD and HG conducted the study, analyzed the data, and prepared and edited the original draft. FS, YL, and MB helped in sampling and measuring. JY and HL participated in managing the experiment. KS helped in writing and editing the manuscript. All authors contributed to the article and approved the submitted version.
