# Supplementary material for: Fertilizer 15N balance in a soybean–maize–maize rotation system based on a 41-year long-term experiment in Northeast China
Source: Front Plant Sci. 2023 Jan 30;14:1105131. doi: 10.3389/fpls.2023.1105131 (PMC9922693; doi:10.3389/fpls.2023.1105131)
Supplement: Supplementary file 1 [file Table_1.docx]

***Supplementary Material***

**Supplementary Tables**

Table S1 Application rates of chemical and organic fertilizers in the soybean and maize seasons in the 41-year long-term experiment.

| Treatments | Chemical fertilizer rates (kg ha^–1^) | | | | | | | | | Pig manure (t ha^–1^) | | |
| --- | --- | --- | --- | --- | --- | --- | --- | --- | --- | --- | --- | --- |
|  | N | | | P_2_O_5_ | | | K_2_O | | |  |  |  |
|  | Soybean | | Maize | Soybean | | Maize | Soybean | | Maize | Soybean | | Maize |
| N | 30 | 120 | | 0 | 0 | | 0 | 0 | | 0 | 0 | |
| NP | 30 | 120 | | 90 | 60 | | 0 | 0 | | 0 | 0 | |
| NPK | 30 | 120 | | 90 | 60 | | 90 | 60 | | 0 | 0 | |
| MN | 30 | 120 | | 0 | 0 | | 0 | 0 | | 0 | 13.5 | |
| MNP | 30 | 120 | | 90 | 60 | | 0 | 0 | | 0 | 13.5 | |
| MNPK | 30 | 120 | | 90 | 60 | | 90 | 60 | | 0 | 13.5 | |

N: chemical N fertilizer; NP: chemical N and P fertilizer; NPK: chemical N, P, and K fertilizer; MN: chemical N fertilizer combined with manure; MNP: chemical N and P fertilizer combined with manure; MNPK: chemical N, P, and K fertilizer combined with manure. The average concentration of nutrients in pig manure during the 41 years is 14.40% for organic matter, 0.72% for total N, 0.87% for total P (P_2_O_5_), and 0.99% for total K (K_2_O).

Table S2 Chemical properties of the 0–20 and 20–40 cm soil layers at sowing in 2017.

| Layer (cm) | Treatment | Organic matter  (g kg^–1^) | Total N  (g kg^–1^) | Available N  (mg kg^-1^) | Olsen-P (mg kg^–1^) | Available K  (mg kg^–1^) | pH (H_2_O) | Mineral N | |
| --- | --- | --- | --- | --- | --- | --- | --- | --- | --- |
|  |  |  |  |  |  |  |  | (mg kg^–1^) | |
|  |  |  |  |  |  |  |  | NO_3_^–^-N | NH_4_^+^-N |
| 0–20 | N | 16.5 (0.61) b | 0.79 (0.01) b | 106.2 (2.5) c | 4.2 (0.2) e | 94.9 (1.1) d | 5.32 (0.05) c | 9.1 (1.9) b | 0.4 (0.1) a |
|  | NP | 15.8 (0.29) b | 0.75 (0.00) b | 106.2 (2.8) c | 28.3 (0.5) d | 81.5 (2.4) e | 5.41 (0.02) c | 7.1 (1.0) b | 0.5 (0.01) a |
|  | NPK | 17.2 (0.34) b | 0.84 (0.01) b | 108.9 (1.8) c | 27.4 (1.2) d | 96.5 (0.7) cd | 5.38 (0.01) c | 7.9 (1.1) b | 0.3 (0.04) a |
|  | MN | 22.7 (0.53) a | 1.05 (0.03) a | 120.4 (0.3) b | 75.4 (0.4) c | 103.4 (4.1) c | 5.65 (0.07) b | 31.7 (0.8) a | 0.4 (0.1) a |
|  | MNP | 22.1 (0.82) a | 1.03 (0.06) a | 134.9 (2.5) a | 164.6 (2.7) a | 118.2 (2.0) b | 5.76 (0.04) a | 28.2 (4.6) a | 1.1 (0.5) a |
|  | MNPK | 22.4 (1.01) a | 1.06 (0.04) a | 123.6 (1.8) b | 153.0 (2.1) b | 152.2 (3.6) a | 5.63 (0.02) b | 26.6 (0.6) a | 1.1 (0.1) a |
| 20–40 | N | 11.1 (0.58) b | 0.61 (0.02) ab | 79.5 (2.5) a | 5.0 (0.2) c | 110.7 (1.5) a | 5.71 (0.02) c | 7.0 (1.2) b | 0.4 (0.07) a |
|  | NP | 11.3 (0.55) b | 0.54 (0.05) b | 70.2 (0.6) b | 7.2 (0.5) c | 92.6 (1.9) c | 5.61 (0.02) c | 5.6 (1.7) b | 0.4 (0.04) a |
|  | NPK | 12.5 (0.59) b | 0.59 (0.04) ab | 79.6 (0.6) a | 17.6 (2.0) b | 102.3 (2.2) b | 5.49 (0.03) d | 6.1 (1.1) b | 0.3 (0.05) a |
|  | MN | 12.0 (0.80) b | 0.56 (0.02) ab | 77.9 (2.2) a | 14.3 (0.6) b | 102.4 (2.5) b | 5.91 (0.06) b | 9.3 (2.8) ab | 0.4 (0.07) a |
|  | MNP | 14.0 (0.42) a | 0.65 (0.01) a | 84.3 (5.1) a | 36.3 (2.5) a | 94.4 (2.2) c | 6.19 (0.02) a | 15.5 (1.7) a | 0.7 (0.1) a |
|  | MNPK | 14.8 (0.67) a | 0.66 (0.02) a | 83.5 (4.4) a | 37.0 (1.7) a | 102.8 (1.2) b | 6.01 (0.01) b | 14.2 (1.0) a | 0.5 (0.1) a |

N: chemical N fertilizer; NP: chemical N and P fertilizer; NPK: chemical N, P, and K fertilizer; MN: chemical N fertilizer combined with manure; MNP: chemical N and P fertilizer combined with manure; MNPK: chemical N, P, and K fertilizer combined with manure. Values are means (standard error of estimated) (n = 3 replicates). Different lowercase letters in each column denote significant differences (*p* < 0.05) in chemical properties among treatments in the 0–20 and 20–40 cm soil layers in the soybean–maize–maize system according to the LSD test.
